# Stepping back to cope: a moderated mediation model of digital disconnection and online social anxiety

**DOI:** 10.3389/fpsyg.2026.1810079

**Published:** 2026-04-30

**Authors:** Shangqing Cao, Songya Liu, Honghao Kang, Zhao Li

**Affiliations:** 1 School of International Education, South China University of Technology, Guangzhou, China; 2 School of Journalism and Communication, South China University of Technology, Guangzhou, China; 3 Faculty of Arts and Social Sciences, University of New South Wales, Sydney, NSW, Australia; 4 High-tech Industry College, Yiyang Vocational and Technical College, Yiyang, China

**Keywords:** connectedness to nature, digital disconnection, online social anxiety, problematic social media use, social media overload

## Abstract

**Introduction:**

Digital disconnection has been proposed as a strategy for mitigating digital strain; however, its psychological correlates and boundary conditions remain insufficiently understood.

**Methods:**

Drawing on survey data from 613 Chinese young adults, this study examines how digital disconnection is associated with online social anxiety using a moderated chain mediation model. Structural equation modeling was employed to test the hypothesized relationships.

**Results:**

Digital disconnection was associated with lower levels of online social anxiety. This association was linked to a sequential pathway whereby lower problematic social media use was associated with lower perceived social media overload, which in turn was associated with lower online social anxiety. Connectedness to nature differentially shaped these processes. At the behavioral level, higher connectedness to nature strengthened the negative association between digital disconnection and problematic social media use. At the cognitive level, however, a paradoxical pattern emerged, whereby stronger connectedness to nature was associated with higher perceived social media overload in the context of digital disconnection.

**Discussion:**

By disentangling the behavioral and cognitive processes associated with digital disconnection, this study extends current understanding of digital well-being beyond simple use–outcome associations. The findings highlight the importance of considering individual differences in psychological resources when designing interventions to promote healthier digital media engagement.

## 1 Introduction

Digital technologies have become deeply woven into everyday life, shaping how individuals communicate, work, and maintain social relationships. While constant connectivity offers convenience and efficiency, it has also intensified concerns surrounding excessive digital engagement. The condition of being “permanently online and permanently connected” has increasingly been associated with a range of unintended consequences, drawing scholarly attention to the ambivalent nature of digital technologies. As Natale and Treré (2020) argue, the technology paradox re-mains highly relevant: the same technologies that enhance daily functioning may, when overused or misused, contribute to pathological dependence. A growing body of research has documented the psychological and cognitive costs of over connection, including attention deficits, diminished social sensitivity, and symptoms of technology-related addiction (Small et al., 2020). At the same time, issues such as information overload, heightened privacy risks, and the blurring of boundaries between online and offline life place sustained psychological pressure on individuals. Rather than representing isolated problems, these challenges have become embedded in everyday digital experiences, raising pressing questions about mental health and digital well-being in contemporary societies.

From the perspective of self-determination theory, individuals are not passive recipients of environmental pressures but are inherently motivated to regulate their behavior in pursuit of psychological growth and well-being (Ryan and Deci, 2000). In response to the strains associated with excessive digital engagement, some users have begun to critically reassess their patterns of technology use. This reflective turn has given rise to a range of practices aimed at deliberately limiting digital connectivity. Concepts such as digital disconnection, digital detox, and digital minimalism have thus emerged as proactive strategies through which individuals seek to recalibrate their relationship with digital media (Schmuck, 2020). Among these related approaches, the present study focuses specifically on digital disconnection, conceptualized as a voluntary and self-regulated reduction in digital engagement.

Although digital disconnection is commonly regarded as a beneficial approach to enhancing digital well-being, empirical findings in this area remain mixed. While a number of studies suggest that reducing digital engagement may alleviate stress and improve psychological outcomes, other evidence points to less optimistic effects. For example, Min et al. (Jiang et al., 2023) found that when individuals are unable to sustain social connections through mobile devices, depressive symptoms may actually intensify. Similarly, disabling smartphone notifications does not necessarily lead to reduced use or fewer checking behaviors; instead, users may experience heightened fear of missing out (FOMO) (Dekker et al., 2025). These inconsistent findings suggest that the effects of digital disconnection cannot be fully understood without closer attention to the psychological processes through which it operates.

In particular, existing research has yet to clearly explain how digital disconnection translates into improvements in individual well-being, or under what conditions such benefits are most likely to occur. Rather than producing uniform outcomes, digital disconnection may trigger a series of behavioral and cognitive changes that jointly shape users’ psychological experiences. Understanding these pathways is therefore critical for evaluating when and how digital disconnection can function as an effective coping strategy.

Within the Chinese research context, digital disconnection warrants particular attention due to the distinctive structure of the local digital ecosystem and its broader socio-cultural pressures. Unlike many Western contexts where social media platforms serve relatively bounded communicative functions, China’s digital environment is characterized by the dominance of “super-apps,” most notably WeChat, which integrates social networking with payment systems, work coordination, transportation, travel services, and everyday life management. In addition, the competitive and performance-oriented social climate often described as “involution” further amplifies pressures to remain constantly available, responsive, and socially visible in digital spaces. Within this context, digital disconnection is not merely a matter of individual preference but a psychologically consequential practice shaped by structural and cultural constraints. Prior studies have largely emphasized qualitative discussions of users’ motivations, conceptualizations, and antecedents of digital disconnection. Empirical investigations into its post-disconnection effects remain relatively limited, and findings derived from Western contexts have not been systematically examined within Chinese social and cultural settings. Addressing this gap, the present study focuses on young social media users in China aged 18–35 and situates digital disconnection within their everyday media practices. A chain mediation model is employed to examine the sequential pathways through which digital disconnection is associated with online social anxiety. In addition, the study considers connectedness to nature—a dispositional tendency reflecting individuals’ perceived closeness to the natural environment—as a potential moderating factor that may shape these post-disconnection processes.

Accordingly, this study addresses two research questions. First, how does digital disconnection influence online social anxiety among young users? Second, does connectedness to nature moderate the mediating pathways through which digital disconnection affects online social anxiety? By addressing these questions, the study contributes to a more nuanced understanding of how psychological resources and self-regulatory behaviors interact to mitigate the adverse consequences of excessive digital connectivity.

## 2 Literature review

### 2.1 Digital disconnection and online social anxiety

Digital disconnection refers to individuals’ intentional or unintentional reduction of engagement with online media and digital devices. Early discussions of digital disconnection largely framed it as a form of disadvantage or exclusion, reflecting limited access to information and communication technologies or avoidance driven by technophobia (Selwyn, 2003; Hesselberth, 2018). In such contexts, disconnection was primarily passive and externally imposed rather than self-initiated.

As digital technologies have become deeply integrated into everyday social and psychological functioning, concerns surrounding excessive connectivity have grown increasingly salient. In response, digital disconnection has gradually been reframed as a voluntary and proactive behavior through which individuals seek to regulate their media use. Contemporary forms of digital disconnection often involve deliberate practices such as limiting time spent on digital media, disabling notifications, or temporarily withdrawing from online platforms (Kaun, 2021; Nguyen et al., 2022). Rather than signaling deficiency, these behaviors reflect an attempt to manage engagement intensity and restore a sense of control over one’s digital environment.

Importantly, given the central role of digital media in shaping social interaction and identity, digital disconnection in the present study does not imply complete withdrawal from online life. Instead, it is conceptualized as a relative and self-regulated reduction in digital engagement, grounded in individuals’ awareness of their own psychological needs and boundaries (Geber et al., 2023). This conceptualization provides a foundation for examining how digital disconnection may influence online social anxiety as a form of adaptive self-regulation.

Digital disconnection fundamentally reflects the relationship between humans and technology, constituting a mode of media engagement. Substantial research confirms the association between social media usage and online social anxiety. Zhang and Zhou (2018) identified a significant positive correlation between passive social media use and social anxiety. A plausible explanation suggests that during passive social media browsing, individuals may experience feelings of inferiority through upward social comparison, thereby inducing negative self-evaluation and emotional distress. Wen et al. (2019) further confirmed that upward social comparison fully mediates the relationship between passive social media use and online social anxiety, suggesting upward comparison may influence self-evaluation anxiety. Stevic (2024) challenged the dichotomy between active and passive social media use, further revealing a long-term reciprocal relationship between active public social media engagement and content-sharing pressure. This suggests that publicly posting content on social media may create a self-reinforcing cycle of pressure. Evidently, whether through active or passive social media use, individuals may experience anxiety and pressure across multiple dimensions, including social comparison, self-presentation, and information overload. Against this backdrop, digital disconnection—as a strategy involving the deliberate cessation or reduction of social media exposure—may be viewed as a psychological coping mechanism through which individuals actively disengage from online social pressures and alleviate social anxiety.

Consequently, this study proposes Hypothesis 1.

*Hypothesis 1*: Digital disconnection negatively influences online social anxiety.

### 2.2 The mediating role of problematic social media use

Problematic social media use is commonly defined as excessive and persistent engagement with mobile social media platforms that leads to negative physiological, psychological, and behavioral outcomes, while not meeting the criteria for a clinical disorder (Jiang, 2018a,b). Prior research has consistently identified a strong association between problematic internet use and negative psychological outcomes, including anxiety (Wei et al., 2024). Students who are heavily engaged in social media tend to report higher levels of online social anxiety and depressive symptoms (Zhihong et al., 2022). Wang et al. (2017) further noted that excessive use of social networking platforms may intensify social anxiety due to ongoing social pressure and unmet interpersonal expectations. Taken together, these findings suggest that problematic social media use serves as an important antecedent of online social anxiety.

According to Self-Determination Theory (SDT), individuals are motivated by three basic psychological needs—autonomy, competence, and relatedness. Individuals inherently seek psychological growth and development, possessing an intrinsic drive to address persistent challenges and integrate external experiences with their sense of self (Ryan and Deci, 2000). Digital disconnection, as a deliberate behavioral choice, initially resembles a ritual. It can be understood as an individual’s attempt to regain control over media usage or viewed as a self-regulation strategy. Both approaches positively influence an individual’s self-control capacity, thereby reducing the likelihood of impulsive social media use. Periods of disconnection also allow users to reflect on their habitual patterns of use and to recognize the negative consequences associated with problematic social media consumption. Through this reflective process, externally driven motivations can be internalized, supporting more deliberate and restrained engagement with social media.

Existing empirical evidence further indicates that digital disconnection is associated with a direct reduction in social media use. During intervention periods, participants showed significant decreases in smartphone and social media engagement, and these reductions remained evident even 2 weeks after the intervention concluded (Coyne and Woodruff, 2023). In addition, digital disconnection may promote alternative offline activities and increase opportunities for face-to-face interaction, thereby strengthening social ties and enhancing real-life satisfaction (Varma, 2018; Jiang and Balaji, 2021).

Based on this theoretical and empirical foundation, this study proposes Hypothesis 2.

*Hypothesis 2*: Problematic social media use mediates the negative impact of digital disconnection on online social anxiety.

### 2.3 The mediating role of social media overload

The concept of overload has been adopted in psychological research to describe individuals’ subjective perceptions and cognitive evaluations when environmental demands exceed their information-processing capacity (Saegert, 1973). Within social media contexts, social media overload refers to a negative psychological state resulting from excessive maintenance of online social relationships and ineffective coping with information exchange technologies (Choi and Lim, 2016).

According to Yu et al.’s (2018) expanded stress–stressor–outcome (ESSO) model, online social anxiety can be understood as a stress response emerging from negative online experiences that are triggered by specific stressors. In contemporary digital environments, social media overload represents a pervasive source of stress. The large volume and fragmented nature of online content, repetitive or low-quality information, increasingly complex interpersonal interactions, and irrational expressions and engagements collectively place substantial demands on users’ cognitive resources, often exceeding their capacity to process information effectively (Choi and Lim, 2016). Under such conditions, social media overload is likely to contribute to heightened levels of online social anxiety.

From the perspective of cognitive load theory, individuals possess limited cognitive resources for processing information and completing tasks. When confronted with excessive information and continuous social interaction demands, users must allocate considerable cognitive effort, which can result in mental fatigue. Previous research has shown that the characteristics of social media, such as permanent online connectivity and constant availability, impose sustained pressure on users and increase feelings of overload, whereas digital disconnection helps alleviate stress and fatigue (Syvertsen and Enli, 2020; Nguyen, 2021). By temporarily disengaging from social media, individuals may better manage social demands, restore work–life balance, and reduce social overload, thereby mitigating associated stress responses (Serrano-Puche, 2017; Nguyen, 2023).

Based on these considerations, this study proposes Hypothesis 3.

*Hypothesis 3*: Social media overload mediates the negative impact of digital disconnection on online social anxiety.

### 2.4 Problematic social media use and social media overload

Digital disconnection allows individuals to reconsider the role that social media plays in their daily lives and to adjust their patterns of use in ways that better serve personal needs, rather than contributing to psychological burden (Varma, 2018; Syvertsen and Enli, 2020). From the perspective of cognitive load theory, such adjustment is meaningful because regulating external stimuli helps preserve limited cognitive resources. When excessive engagement with social media is restrained, problematic patterns of use may become less pronounced, which may in turn be associated with a lower likelihood of social media overload.

The ESSO model proposed by Yu et al. (2018) provides further insight into this process by identifying cognitive overload as a key technological stressor associated with negative online psychological experiences. Notably, the model suggests that excessive social media use itself constitutes a source of stress and precedes the development of downstream stressors, including social media overload. Supporting this view, Maier et al. (2015b) found that intensive social media use is a prerequisite for cognitive overload, as cognitive strain tends to emerge only when individuals are deeply and continuously engaged with social media platforms.

In information-rich social media environments, users often find it difficult to maintain attention across ongoing streams of content or to respond effectively to multiple social demands, which increases their susceptibility to overload. In this context, digital disconnection may function as a practical means of reducing problematic social media use, thereby lowering exposure to conditions that foster social media overload.

Accordingly, this study proposes Hypothesis 4: Problematic social media use and social media overload sequentially mediate the relationship between digital disconnection and online social anxiety.

### 2.5 The moderating role of connectedness to nature

Connectedness to nature (CTN) has been conceptualized as a relatively stable individual characteristic reflecting differences in how people perceive and experience their relationship with the natural environment. Drawing on work by scholars such as E. O. Wilson, Mayer, and Frantz, CTN is generally understood as capturing the quality and intimacy of human–nature relationships. Previous studies have shown that connectedness to nature is associated with beneficial cognitive, emotional, and behavioral outcomes. More recently, this concept has begun to attract attention in research on media use and digital behavior, although empirical evidence in this area remains limited.

From the perspective of self-determination theory, the satisfaction of basic psychological needs contributes to psychological well-being and supports more adaptive behavioral patterns. Natural environments have been shown to provide restorative experiences characterized by beauty and tranquility, which can stimulate intrinsic motivation and enhance feelings of autonomy and satisfaction (Baxter and Pelletier, 2019; Hurly and Walker, 2019). As settings for offline activities, natural spaces also create opportunities for face-to-face interaction, thereby supporting relatedness needs. In addition, interaction with nature may foster a sense of place and belonging even in the absence of direct social engagement. Together, these processes suggest that connectedness to nature may help regulate media use by fulfilling underlying psychological needs, potentially reducing reliance on social media.

Attention Restoration Theory (ART) offers further insight into the role of nature in alleviating cognitive strain. According to ART, natural environments contain stimuli that capture attention in a bottom-up manner, allowing directed, top-down attention to recover (Berman et al., 2008). Exposure to nature can therefore support the restoration of cognitive resources without requiring sustained effortful processing, which may help offset cognitive overload associated with intensive social media use. Moreover, engagement with natural environments has been linked to reductions in depression, anxiety, and stress, as well as improvements in overall health (Dean et al., 2018). By alleviating psychological strain and enhancing emotional states, these benefits may further reduce susceptibility to cognitive overload.

Thus, this study proposes Hypothesis 5: Connectedness to nature moderates the relationship between digital disconnection behaviors and both problematic social media use and social media overload.

## 3 Materials and methods

### 3.1 Participants and procedure

This study used a questionnaire-based survey design. Prior to the main data collection, a pilot study was conducted to assess the clarity, reliability, and validity of the measurement items. The pilot questionnaire was distributed online through social media platforms using a combination of random recruitment and snowball sampling, resulting in 169 valid responses. Based on feedback and statistical results from the pilot test, several items were revised or removed to improve measurement accuracy. In addition, baseline measures of anxiety were included, items distinguishing different motivations for digital disconnection were added, and the sequence of questionnaire items was adjusted. These revisions led to further refinement of variable descriptions and wording, resulting in the final version of the questionnaire.

The formal survey was administered primarily through online communities and social media platforms, including Weibo, Douban, WeChat, and Xiaohongshu. A mixed sampling strategy combining random sampling and snowball sampling was employed. Procedurally, several steps were taken to reduce potential common method bias. Participants were informed that the survey was anonymous and voluntary, and that there were no right or wrong answers. These measures were intended to reduce evaluation apprehension and encourage more candid responses. In total, 883 questionnaires were collected. After data screening, responses that did not meet the target age criteria, failed attention-check items, or were completed in an unreasonably short time were excluded. The final sample consisted of 613 valid questionnaires, which were used for subsequent analyses.

The final sample included 234 male and 379 female respondents, yielding a male-to-female ratio of 1:1.619 and indicating a slight predominance of female participants. Regarding educational background, 63.0% of respondents reported holding a bachelor’s degree or higher. By occupation, students constituted the largest group (32.3%), followed by company employees and freelancers. Participation from service industry workers, manual laborers, and public servants was relatively low in this survey. In terms of age, 351 respondents (57.3%) were between 18 and 25 years old, reflecting a strong representation of Generation Z. The 26–30 age group included 153 respondents, and 109 respondents were aged 31–35.

In the demographic section, participants reported gender, age, highest level of education, and occupational status. The author first conducted independent samples t-tests and one-way ANOVA on these four variables to examine whether demographic factors exerted significant influence. Based on these results, gender was included as a control variable in subsequent analyses. In addition, baseline anxiety and disconnection motivation were controlled to account for their well-established associations with social media use and psychological outcomes, thereby isolating the unique effects of the focal constructs.

Participation in this study was voluntary and anonymous. Before completing the online questionnaire, participants were informed of the purpose of the study and their right to withdraw at any time. Electronic informed consent was obtained at the beginning of the survey. In accordance with institutional practice for anonymous, minimal-risk questionnaire research, formal ethics committee approval was not required for this study.

### 3.2 Measures

#### 3.2.1 Digital disconnection behavior

Digital disconnection behavior was assessed using an adapted scale based on the Digital Disconnection Strategies Questionnaire (Nguyen et al., 2022) and measures of digital disconnection behavior proposed by (Geber et al., 2023). The original pool contained 15 items. Following confirmatory factor analysis, one item was removed due to low factor loading, resulting in a final 14-item scale. Participants responded on a five-point Likert scale. A sample item is “When I engage in other activities, I put my digital devices aside.” Higher scores indicate greater engagement in digital disconnection behavior. Cronbach’s *α* was 0.91.

#### 3.2.2 Problematic social media use

Problematic social media use was measured using the Problematic Mobile Social Media Use Assessment Questionnaire (Jiang, 2018a, 2018b). The original instrument includes 20 items. In the present study, six items were removed following confirmatory factor analysis due to low standardized loadings, yielding a final 14-item scale. Participants rated items on a five-point Likert scale. A sample item is “I unconsciously and frequently scroll through mobile apps or check social media feeds daily, losing track of how many times I do so.” Higher scores indicate greater problematic social media use. Cronbach’s *α* was 0.88.

#### 3.2.3 Social media overload

Social media overload was measured using an adapted scale based on prior research on information overload, social overload, and functional overload (Koroleva et al., 2011; Maier et al., 2015b; Zhang et al., 2016, 2017). The scale includes 8 items capturing users’ perceived cognitive and functional burden associated with social media use. In the present study, the three dimensions were modeled as a single higher-order construct reflecting overall social media overload. Participants responded on a five-point Likert scale ranging from 1 (strongly disagree) to 5 (strongly agree). A sample item is “Features on social media unrelated to my primary purpose of use often annoy me.” Higher scores indicate greater perceived overload. Cronbach’s *α* was 0.90.

#### 3.2.4 Online social anxiety

Online social anxiety was measured using the Social Anxiety Scale for Social Media Users (SAS-SMU; Alkis et al., 2017). The original scale contains 21 items; however, six items were removed based on confirmatory factor analysis due to low factor loadings, resulting in a final 15-item scale. Responses were recorded on a five-point Likert scale ranging from 1 (strongly disagree) to 5 (strongly agree). A sample item is “I worry that other users on social media will find my behavior embarrassing.” Higher scores indicate greater levels of online social anxiety. In this study, Cronbach’s *α* was 0.94.

#### 3.2.5 Connectedness to nature

Connectedness to nature was measured using the Connectedness to Nature Scale (CNS; Mayer and Frantz, 2004). The original scale contains 14 items. In the present study, three items were removed based on confirmatory factor analysis due to low standardized factor loadings, resulting in a final 11-item version. Participants responded on a five-point Likert scale ranging from 1 (strongly disagree) to 5 (strongly agree). A sample item is “I often feel at one with the natural world around me.” Higher scores indicate stronger connectedness to nature. Cronbach’s α for the revised scale was 0.92.

### 3.3 Validity assessment

Because several scales were adapted for the present research context, item refinement was conducted prior to the final analyses. Items with consistently low factor loadings, conceptual redundancy, or poor fit in the measurement model were removed based on both statistical criteria and theoretical interpretability. The final retained items for each construct were subjected to confirmatory factor analysis, and all retained scales demonstrated acceptable reliability and construct validity. To improve transparency, detailed information on the retained and removed items is provided in the Supplementary Material.

Confirmatory factor analysis (CFA) was conducted to examine the structural validity of the measurement model. As shown in Table 1, the model demonstrated an acceptable fit to the data, with a χ^2^/df value of 2.078 (below the recommended threshold of 3). In addition, the RMSEA value was below the critical value of 0.08, while the incremental fit indices (IFI, TLI, and CFI) all exceeded 0.90. Taken together, these results indicate that the proposed measurement model exhibits good overall model fit.

**Table 1:** Overall model fit indices for the measurement model.

Model fit indices				
X2/df	RMSEA	IFI	TLI	CFI
2.078	0.042	0.919	0.915	0.919

Discriminant validity was assessed using the Fornell–Larcker criterion. The square roots of the average variance extracted (AVE) for digital disconnection, problematic social media use, social media overload, online social anxiety, and connectedness to nature were 0.644, 0.761, 0.730, 0.710, and 0.715, respectively. All inter-construct correlation coefficients were lower than the corresponding square roots of AVE, providing evidence of satisfactory discriminant validity among the study variables (see Table 2).

**Table 2:** Discriminant validity of the measurement scales.

Constructs	Digital disconnection	Problematic social media use	Social media overload	Online social anxiety	Connectedness to nature
Digital disconnection	0.644				
Problematic social media use	−0.297	0.761			
Social media overload	−0.166	0.737	0.730		
Online social anxiety	−0.365	0.759	0.608	0.710	
Connectedness to nature	0.451	−0.192	−0.119	−0.307	0.715

## 4 Results

### 4.1 Correlation analysis and common method bias assessment

The correlation matrix among the study variables is presented in Table 3. The results reveal that all key variables are significantly correlated with one another (*p* < 0.01). Because all focal variables were collected through self-report questionnaires at a single time point, common method bias was considered. Harman’s single-factor test was first conducted. The results showed that the first unrotated factor accounted for 33.267% of the total variance, which was below the commonly referenced 40% threshold. This suggests that common method bias was unlikely to fully account for the observed associations.

**Table 3:** Descriptive statistics and correlations among study variables.

Variables	1	2	3	4	5
1 DD	1				
2 PSMU	−0.331***	1			
3 SMO	−0.205***	0.806***	1		
4 OSA	−0.432***	0.759***	0.694***	1	
5 CTN	0.751***	−0.266***	−0.187***	−0.435***	1

All variables were standardized prior to analysis. *p* < 0.10, *p* < 0.05, *p* < 0.01.

### 4.2 Path analysis

Path analysis within the structural equation modeling (SEM) framework was employed to examine the hypothesized relationships among the primary variables. The results of the path analysis indicate that all specified paths in the model are statistically significant (*p* < 0.01 see Table 4).

**Table 4:** Summary of standardized path coefficients for the structural model.

X	→	Y	Standardized coefficients	S.E.	C.R.	*p*
DD	→	PSMU	−0.463	0.057	−8.113	***
PSMU	→	SMO	0.798	0.05	15.918	***
DD	→	SMO	0.109	0.031	3.483	***
SMO	→	OSA	0.283	0.085	3.346	***
DD	→	OSA	−0.268	0.039	−6.868	***
PSMU	→	OSA	0.487	0.079	6.151	***

All variables were standardized prior to analysis. *p* < 0.10, *p* < 0.05, *p* < 0.01.

### 4.3 Chain mediation effects of problematic social media use and social media overload

**Figure 1:**
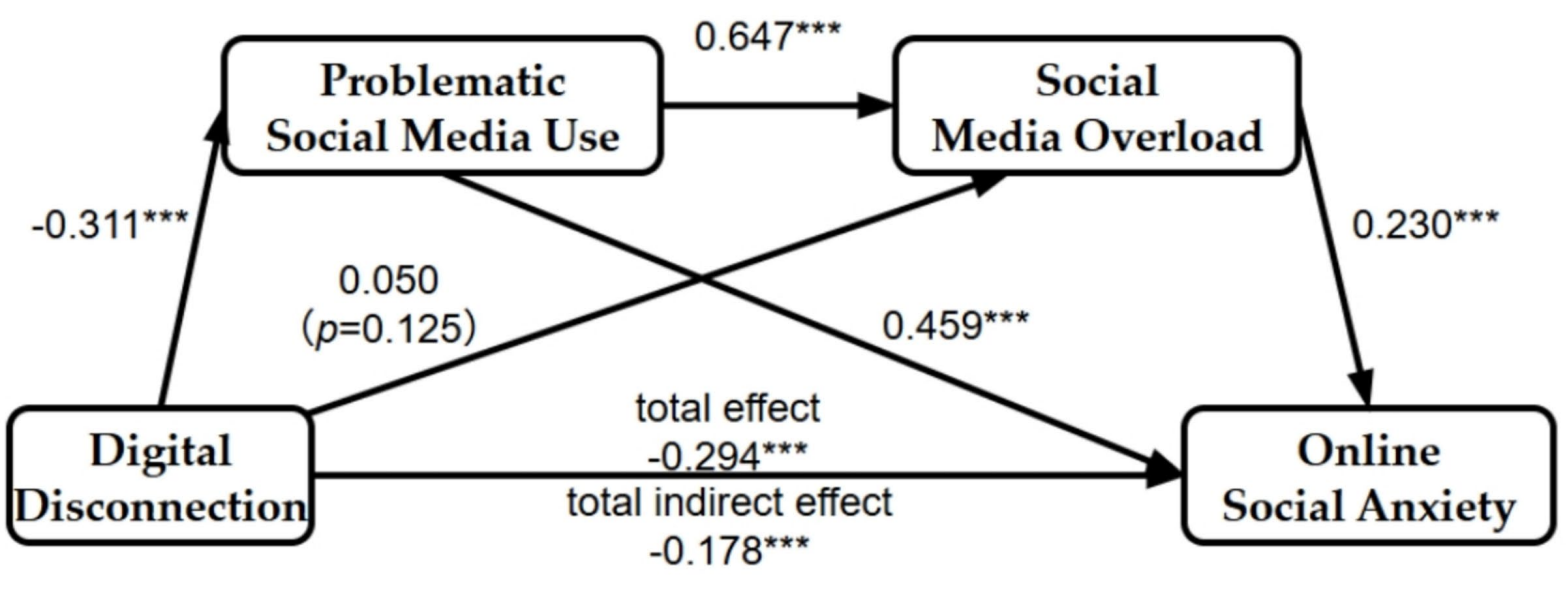
Chain mediation model of problematic social media use and social media overload.

To examine the sequential mediating roles of problematic social media use and social media overload, regression-based mediation analyses were conducted using the PROCESS macro (version 4.1) for SPSS, specifically Model 6. As shown in Figure 1, the associations between digital disconnection and online social anxiety, problematic social media use and social media overload, as well as problematic social media use and social media overload on online social anxiety were all statistically significant (*p* < 0.01). However, within the PROCESS Model 6 analysis, the association between digital disconnection and social media overload was not statistically significant.

It should be noted that this path showed slightly different significance patterns across analytic approaches. In the SEM model, the path from digital disconnection to social media overload reached statistical significance, whereas in the PROCESS Model 6 analysis it did not remain significant when problematic social media use and control variables were simultaneously included. This difference may reflect methodological distinctions between the two approaches, as SEM estimates latent-variable relationships while accounting for measurement error, whereas PROCESS relies on observed composite scores in regression-based estimation. Substantively, this pattern suggests that the association between digital disconnection and social media overload may be comparatively weaker and may operate more meaningfully through problematic social media use rather than as a stable independent direct pathway.

To further examine the indirect associations, bootstrap analyses were conducted. The significance of each indirect effect was determined based on whether the 95% confidence interval included zero. As shown in Table 5, this mediating effect encompasses three pathways. The interval for “digital disconnection → problematic social media use → online social anxiety” did not include zero, indicating a significant indirect association through problematic social media use. The “digital disconnection → social media overload → online social anxiety” interval included zero, indicating that this indirect association was not statistically significant. The “digital disconnection → problematic social media use → social media overload → online social anxiety” interval excluded zero, supporting a significant chain indirect association.

**Table 5:** Results of the chain mediation analysis for problematic social media use and social media overload.

Paths	Effect	Boot SE	BootLLCI	BootULCI
Total indirect effect	−0.178	0.031	−0.240	−0.120
Ind1 DD → PSMU → OSA	−0.143	0.028	−0.199	−0.092
Ind2 DD → SMO → OSA	0.011	0.009	−0.004	0.032
Ind3 DD → PSMU → SMO → OSA	−0.046	0.014	−0.076	−0.023

### 4.4 Moderating effect of connectedness to nature

A moderated mediation analysis was conducted using Model 84 of the PROCESS macro (version 4.1) in SPSS to examine whether connectedness was associated with variation in the indirect pathways linking digital disconnection to downstream outcomes. The results showed that connectedness to nature had a marginally significant moderating association with the relationship between digital disconnection and problematic social media use. The interaction between digital disconnection and connectedness to nature negatively predicted problematic social media use (*β* = −0.0942, *p* = 0.0534), indicating that the association between digital disconnection and problematic social media use varied depending on individuals’ levels of connectedness to nature.

**Figure 2:**
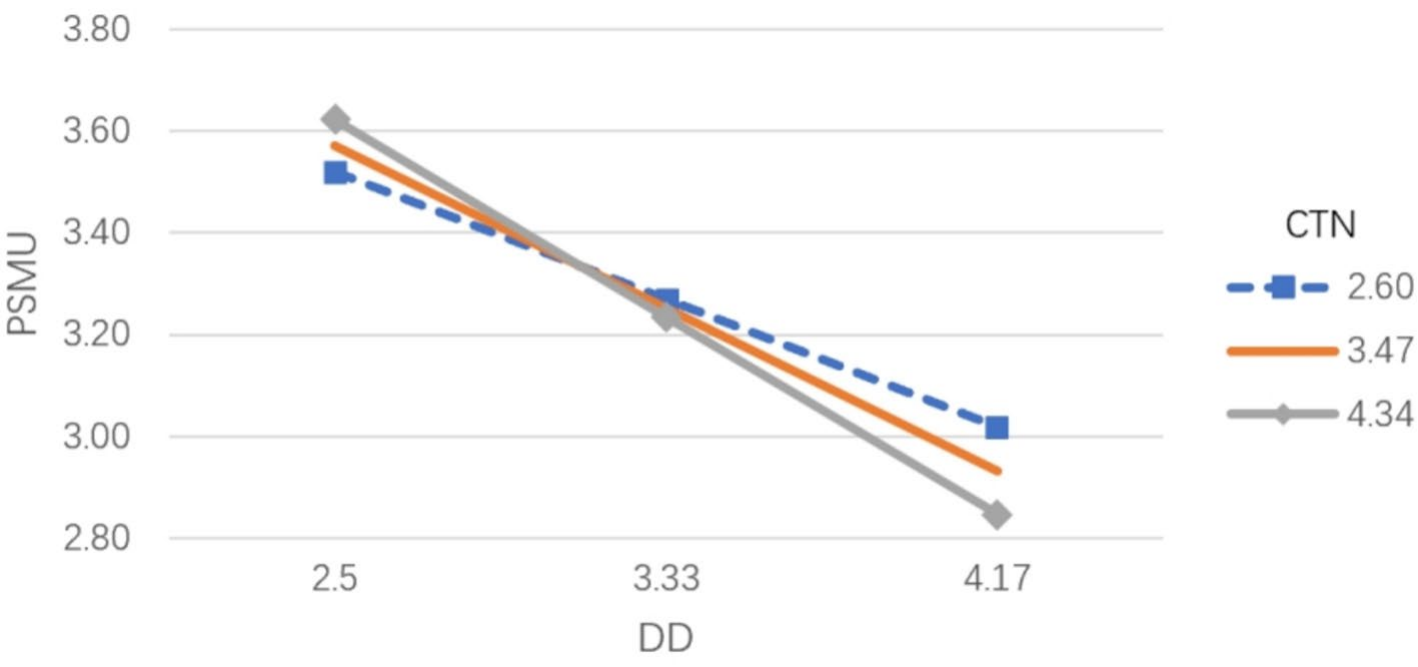
Moderating effect of connectedness to nature on the relationship between digital disconnection and problematic social media use.

**Figure 3:**
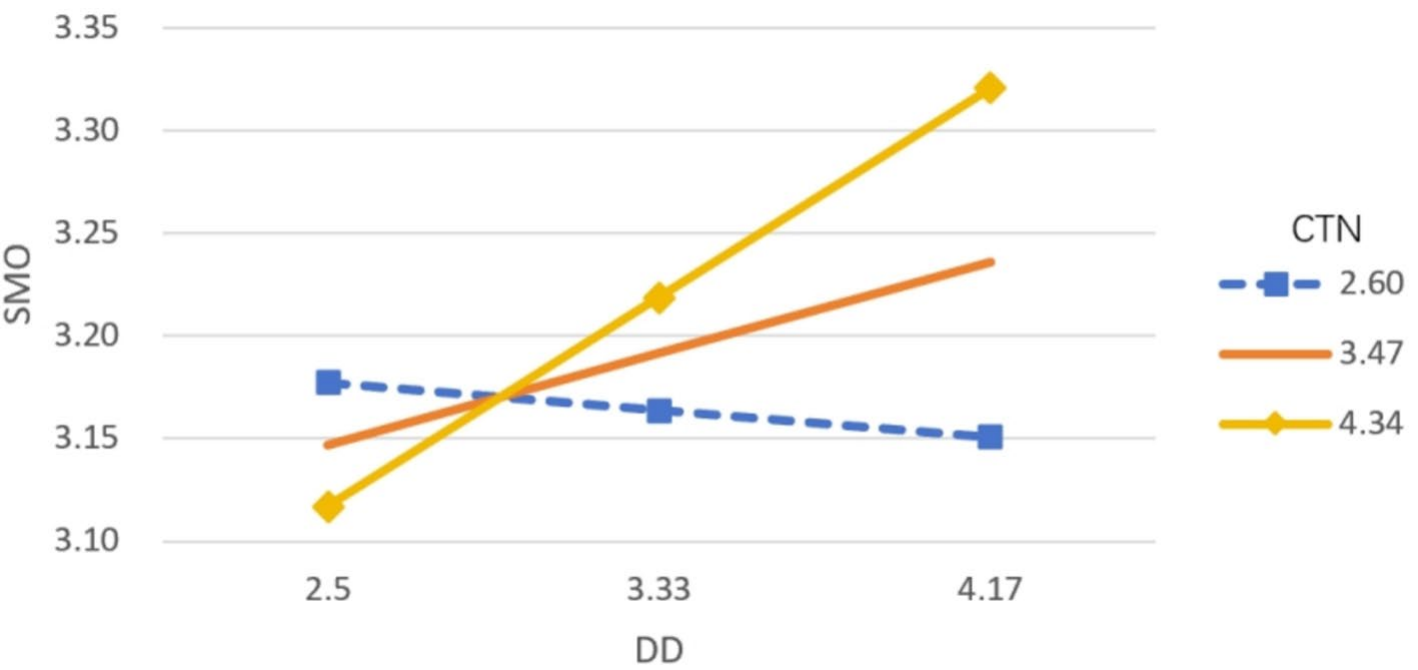
Moderating effect of connectedness to nature on the relationship between digital disconnection and social media overload.

In the model examining social media overload, connectedness to nature also moderated the association involving digital disconnection, but the direction of this interaction differed. Specifically, the interaction term positively predicted social media overload (*β* = 0.0792, *p* < 0.05). To further interpret these interactions, a simple slope analysis was performed (Figures 2, 3). The analysis indicated that the negative association between digital disconnection and problematic social media use became stronger as connectedness to nature increased (bsimple low = −0.301, *p* < 0.001; bsimple high = −0.470, *p* < 0.001). For social media overload, the association with digital disconnection was non-significant at low levels of connectedness to nature (*p* = 0.761), showed a positive but non-significant trend at moderate levels (*β* = 0.053, *p* = 0.206), and reached statistical significance at high levels (*β* = 0.122, *p* < 0.05).

### 4.5 Control variables

Baseline anxiety was positively associated with the focal variables across models. Including it as a control variable allowed the analyses to account for its potential association with the outcomes. Proactive disconnection motivation was positively associated with problematic social media use (*β* = 0.1067, *p* < 0.05) and negatively associated with online social anxiety (*β* = −0.1146, *p* < 0.001). This pattern suggests that proactive disconnection motivation may be linked to more frequent engagement-related tendencies while also being associated with lower levels of anxiety.

Passive disconnection motivation was positively associated with both problematic social media use (*β* = 0.3822, *p* < 0.001) and social media overload (*β* = 0.1633, *p* < 0.001), but it was not significantly associated with online social anxiety. This likely indicate that passive disconnection motivation is more closely related to problematic engagement and perceived overload than to anxiety directly.

Gender was also significantly associated with problematic social media use (*β* = 0.3152, *p* < 0.001), with females reporting higher levels than males. Its association with social media overload was marginally significant, suggesting a possible gender-related difference in this domain. However, gender was not significantly associated with online social anxiety.

## 5 Discussion

### 5.1 Main findings

The present study employed a moderated chain mediation framework to clarify how digital disconnection is associated with online social anxiety through behavioral and cognitive pathways. Overall, the findings suggest that digital disconnection is linked to lower levels of online social anxiety, both directly and indirectly, primarily through its association with problematic social media use. Social media overload, although positively associated with anxiety, did not function as an independent mediator. Instead, it appears to operate downstream of behavioral dependence, suggesting that problematic use may represent a more proximal pathway linking digital engagement to emotional outcomes.

The negative association between digital disconnection and online social anxiety highlights the potential psychological significance of intentional disengagement. From a self-determination theory perspective, digital disconnection may be understood as an autonomous behavioral choice. When individuals perceive that continuous connectivity disrupts their emotional equilibrium, choosing to disconnect may restore a sense of control over time allocation, social responsiveness, and personal boundaries. This interpretation is consistent with the observed association between digital disconnection and lower anxiety.

This pattern may be particularly relevant for young people, whose social identities and emotional expression are deeply embedded in digital environments. Within an “always-on” culture characterized by rapid response expectations and constant visibility, social media participation can become intertwined with performance concerns and evaluation anxiety. In this context, digital disconnection is not merely a temporary coping strategy, but an intentional effort toward psychological self-regulation and media boundary management.

Among the mediating variables, problematic social media use emerged as the most robust pathway. This also aligns with previous findings that digital addiction tendencies are positively associated with social anxiety (Ime et al., 2024). Unlike social media overload, which reflects a cognitive appraisal of excessive information and social demands, problematic use captures behavioral dependence and habitual engagement patterns. This distinction helps explain why the association between digital disconnection and anxiety was more strongly linked to behavioral adjustment than to immediate cognitive relief. Even when time spent online decreases, perceptions of unfinished interactions, unread messages, or social obligations may persist. Prior research has conceptualized overload as a “perceptual burden” that accumulates structurally and may exhibit latency effects: individuals’ perceptions of information volume, social interaction pressures, and response obligations contribute to this sense of overload, rather than mere frequency or duration of platform use (Maier et al., 2015a) and even after disconnection, individuals may still worry about missed information or social opportunities (Koroleva et al., 2010). Thus, overload is less likely to function as a primary first-stage mechanism and more likely to emerge downstream of behavioral dysregulation, particularly problematic social media use.

Importantly, the findings support a chained behavioral–cognitive–emotional pathway. Digital disconnection was associated with lower problematic use, which in turn was associated with lower perceived overload and subsequently lower online social anxiety. This sequence offers a more differentiated account of how intentional disengagement may relate to escalating psychological strain in digital contexts.

Connectedness to nature further shaped these processes in nuanced ways. Higher levels of nature connectedness strengthened the negative association between digital disconnection and problematic use, suggesting that psychological resources derived from nature may be linked to stronger self-regulatory capacity and emotional resilience. Nature may serve as a non-digital substitute for restoration, reducing reliance on virtual social validation and supporting psychological self-sufficiency. However, the moderating pattern was not uniform across pathways. Among individuals with stronger connectedness to nature, digital disconnection was associated with a slight increase in perceived social media overload. This counterintuitive pattern suggests that psychological resources may operate differently across behavioral and cognitive domains. While exposure to nature has been associated with cognitive recovery, it may also heighten sensitivity to redundant information. Gu et al. (2023) found that engagement with natural scenery may enhance cognitive evaluation, which could make highly nature-connected individuals more aware of social expectations and interaction imbalances (Capaldi et al., 2014). From a FOMO perspective, temporary disengagement may heighten concerns about missed updates or social feedback, producing a short-term rebound effect (Przybylski et al., 2013). For highly sensitive individuals, digital disconnection may function not only as a restorative practice but also as an emotion-eliciting condition. These findings underscore the complexity of psychological boundary conditions in digital well-being processes and highlight the importance of considering individual differences when evaluating the associations linked to digital detox behaviors (Nguyen, 2022).

Taken together, the results contribute to a more differentiated understanding of how digital health behaviors are associated with mental health outcomes. Rather than assuming that reduced digital exposure uniformly alleviates distress, the present findings suggest that behavioral dependence may represent the more immediate intervention target, whereas cognitive overload may reflect a secondary, context-sensitive process. By integrating behavioral, cognitive, and emotional dimensions within a moderated framework, this study offers a more nuanced account of how intentional digital disconnection may be linked to psychological well-being.

### 5.2 Theoretical implications

This study offers several theoretical contributions. One important contribution is that it situates the analysis within the context of social media use, showing how users employ digital disconnection to restore autonomy and manage psychological burdens amid information overload and emotional challenges. By doing so, it extends self-determination theory and related frameworks to emerging digital behaviors in contemporary social media environments. In addition, it encourages interdisciplinary dialogue within media audience research, generating new avenues for academic convergence.

Another contribution lies in the construction of a moderated chain mediation model. This provides a novel empirical foundation to address limitations in existing Chinese research on digital disconnection, which has often relied on qualitative methods and offered limited quantitative evidence. The findings also respond to the recent scholarly interest in digital disconnection, digital detox, and digital minimalism.

Finally, the findings suggest that digital disconnection may be associated with lower levels of online social anxiety, highlighting its potential relevance as a positive media use regulation strategy. By integrating behavioral usage and cognitive load pathways within a chained mediation model, it highlights how digital disconnection may be associated with emotional states across both behavioral and cognitive dimensions. The seemingly counterintuitive moderating effect observed in the cognitive pathway offers further theoretical insight into the functionality and boundary conditions of individual psychological resources within contemporary media environments.

### 5.3 Practical implications

For practical implications, the findings suggest that digital disconnection may be a useful strategy for supporting emotional regulation and digital well-being, particularly when it is approached intentionally rather than reactively. To fully benefit from digital disconnection and account for the contradictory effects observed in this study, it is important for users to develop a deep understanding of the purpose of disconnection and to reflect on their internal mental processes before initiating it. At the cognitive level, users may need to reconsider their understanding of “missing out” and “connection.” Cultivating clear information boundaries, self-regulatory awareness, and sensitivity to negative emotions can help clarify whether their behaviors are guided by authentic intentions rather than social anxiety.

From a broader philosophical perspective, digital disconnection is not simply an act of “disconnecting.” It can be seen as a form of psychological “return,” a way to quiet the mind rather than suppress external noise. At the practical or behavioral level, users can plan and set boundaries in advance. Alternative activities can be arranged, and a tiered disconnection approach may help: high-load or highly-inductive platforms can be selectively suspended while essential communication channels remain open. This allows users to balance disconnection with real-world responsibilities. Finally, individuals may integrate mindfulness training into daily life to enhance awareness and acceptance of present experiences, thereby reducing reactive anxiety over missed social updates.

### 5.4 Limitations and future directions

Some limitations should be noted, which future research could address.

First, the present study relied on cross-sectional self-report survey data. Although the proposed mediating and moderating mechanisms were statistically supported, the temporal and causal ordering among the variables cannot be firmly established. Accordingly, the findings should be interpreted as associative rather than causal. Future research could employ longitudinal designs, experience sampling methods, or experimental approaches to strengthen causal inference. In addition, because all focal variables were measured through self-report at a single time point, the findings may be subject to common method bias, self-selection tendencies, and social desirability bias. Although several procedural and statistical steps were taken to reduce these concerns, future studies could further address them by incorporating multi-source data, behavioral trace measures, or time-lagged designs.

Another limitation concerns the sample. The study focused primarily on young adults, which may limit the generalizability of the findings. Future studies should include more diverse populations to examine whether similar patterns are observed across different sociocultural and developmental contexts. Besides, with regard to the bidirectional moderating patterns involving connectedness to nature, this study analyzed the variable only in terms of moderation direction and statistical significance. Future research could examine its multidimensional structure more thoroughly, providing deeper insight into how ecological resources influence psychological moderation in digital environments.

## Data Availability

The raw data supporting the conclusions of this article will be made available by the authors, without undue reservation.
